# Sexual selection moderates heat stress response in males and females

**DOI:** 10.1111/1365-2435.14204

**Published:** 2022-10-27

**Authors:** Maria Moiron, Lennart Winkler, Oliver Yves Martin, Tim Janicke

**Affiliations:** ^1^ Centre d'Écologie Fonctionnelle et Évolutive, CNRS University of Montpellier, EPHE, IRD Montpellier Cedex 05 France; ^2^ Institute of Avian Research Wilhelmshaven Germany; ^3^ Applied Zoology Technical University Dresden Dresden Germany; ^4^ Department of Biology & Institute of Integrative Biology IBZ ETH Zurich Zürich Switzerland

**Keywords:** heatwaves, mating system, reproductive success, thermal stress, *Tribolium castaneum*

## Abstract

1. A widespread effect of climate change is the displacement of organisms from their thermal optima. The associated thermal stress imposed by climate change has been argued to have a particularly strong impact on male reproduction but evidence for this postulated sex‐specific stress response is equivocal.
2. One important factor that may explain intra‐ and interspecific variation in stress responses is sexual selection, which is predicted to magnify negative effects of stress. Nevertheless, empirical studies exploring the interplay of sexual selection and heat stress are still scarce.
3. We tested experimentally for an interaction between sexual selection and thermal stress in the red flour beetle *Tribolium castaneum* by contrasting heat responses in male and female reproductive success between enforced monogamy and polygamy.
4. We found that polygamy magnifies detrimental effects of heat stress in males but relaxes the observed negative effects in females. Our results suggest that sexual selection can reverse sex differences in thermal sensitivity, and may therefore alter sex‐specific selection on alleles associated with heat tolerance.
5. Assuming that sexual selection and natural selection are aligned to favour the same genetic variants under environmental stress, our findings support the idea that sexual selection on males may promote the adaptation to current global warming.

Read the free Plain Language Summary for this article on the Journal blog.

## Graphical abstract

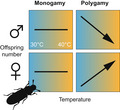

## 1 INTRODUCTION

Ambient temperature is one of the most variable environmental factors that terrestrial organisms face during their lifetimes and is predicted to have profound effects on behaviour, physiology and life‐history traits (Angilletta, 2009; Huey et al., 2012; Tattersall et al., 2012). The Intergovernmental Panel on Climate Change is currently predicting a rise of global surface temperature of 1.2 to 3.0°C until 2060 (IPCC, 2021), which will be likely linked to a higher frequency of extreme weather phenomena such as drought and heatwaves (Coumou & Rahmstorf, 2012; Fischer & Knutti, 2015). Hence, the study of how organisms are affected by and adapt to changes in ambient temperature has become a central topic in ecology and evolutionary biology over the last two decades (Parmesan, 2006; Walsh et al., 2019). Despite the current interest in understanding the effects of climate change on the biosphere, our knowledge on how heat stress affects reproductive performance and the selective processes that modulate heat responses is still limited.

One prominent but still largely unexplored evolutionary process that may affect heat responses is sexual selection (Garcia‐Roa et al., 2020; Pilakouta & Ålund, 2021), here defined as selection arising from competition for mating partners and/or their gametes (Jones & Ratterman, 2009). On an evolutionary scale, sexual selection may select for a higher allocation of resources into current reproduction at the cost of future fitness. In most animal taxa, sexual selection operates more strongly on males (Janicke et al., 2016) for which the so‐called ‘live fast die young’ strategy predicts that males invest less resources into traits facilitating survival including health and stress resilience (Bonduriansky et al., 2008; Tarka et al., 2018), in favour of enhanced reproduction. Therefore, sexual selection is predicted to promote the evolution of higher susceptibility to environmental stress in males compared to females (Hämäläinen et al., 2018). However, formal theoretical work in support of this prediction is scarce and sexual selection on condition‐dependent traits may mitigate the postulated sex differences as explored, for example, for sex‐specific immunocompetence (Stoehr & Kokko, 2006).

In addition to the expected evolution of sex‐specific life histories, sexual selection may amplify the negative effects of environmental stress, because intrasexual competition for mating partners is predicted to favour individuals with superior condition (here defined as the pool of acquired resources that can be allocated towards various fitness routes including traits conveying a higher competitiveness at pre‐ and postcopulatory episodes of sexual selection). The so‐called ‘genic capture’ hypothesis posits that condition is a highly polygenic trait and that sexual selection on condition‐dependent sexual traits favours genetic variants that are also favoured by natural selection in a given environmental context (Rowe & Houle, 1996; Rowe & Rundle, 2021). As a consequence, sexual selection inflates the genetic variance of fitness components (David et al., 2000) and amplifies selection against maladapted genotypes, which manifests in a higher responsiveness to environmental stress in the sex experiencing stronger competition for mates and/or gametes.

Sex‐specific stress responses may, however, not only be driven by sex‐specific sexual selection but can also evolve as a result of ecological character displacement (De Lisle, 2019). Moreover, gamete production is a fundamentally different process in males and females requiring sex‐specific physiologies, which in turn may lead to sex‐specific responses to changing environments. For instance, spermatogenesis is often considered to be particularly sensitive to heat stress—an issue that has been extensively studied in mammals, including humans (Zorgniotti, 1991), and has long been argued to have caused the evolution of external testes (i.e. a scrotum) in several mammalian clades (Lovegrove, 2014; Moore, 1926). More recent work suggests that heat stress can also have a profound impact on male reproductive performance in ectotherms. For instance, sterilizing temperature of males has been found to be a better predictor of the occurrence of 43 *Drosophila* species than lethal temperature (Parratt et al., 2021), suggesting that heat stress on males can impose limits on the geographic distributions of species.

The red flour beetle *Tribolium castaneum*, is a highly tractable study system and an emerging model species for the study of thermal stress responses in ectotherms. In particular, heatwaves of only 5°C above optimal temperatures have been found to compromise sperm production rate and sperm performance, which translated into higher sensitivity to heat stress in males compared to females under monogamy (Sales et al., 2018; Sales et al., 2021). In addition, sperm competition trials revealed that heat‐stressed males obtain a lower paternity share when competing against control males (Sales et al., 2018). However, the role that sexual selection plays as a moderator of sex differences in heat response remains unexplored across ectotherms including *Tribolium* beetles.

In this study we aimed at filling this knowledge gap by testing how sexual selection and thermal stress interact to affect reproductive success of males and females in *T. castaneum*. Specifically, we experimentally manipulated thermal stress by means of simulated heatwaves and permanent heat exposure to contrast heat responses of both sexes in monogamous versus polygamous mating system settings. Using this approach, we investigated the following four questions: (1) Does heat stress affect male and female reproductive success differently? (2) Do the sexes recover differently from heat stress? (3) Does the mating system affect heat responses in males and females? (4) Does the mating system interact with sex differences in heat response?

## 2 MATERIALS AND METHODS

### 2.1 Study system

*Tribolium castaneum* is a highly polygamous species where sexual selection operates especially during postcopulatory episodes in both males and females (Fedina & Lewis, 2008). In this experiment we used a highly outbred strain created by TJ in 2020 (called *MTP1*, Montpellier 1). This strain originates from a full‐diallel mating design, crossing males and females from five strains (i.e. ‘*Ga1*’, ‘*BRZ5*’, ‘*Japan*’, ‘*Abidjan*’ and ‘*Moliste*’) provided by the Agricultural Research Service of the United States Department of Agriculture (USDA, https://www.ars.usda.gov/). All strains have been previously kept at 30°C for hundreds of generations. Each diallel cross was replicated 10 times, which amounted to a total number of 200 pairs. From each pair we collected 10 offspring to start the first generation of the *MT1 strain*. After its creation, the *MTP1* strain has been kept in the laboratory at a population size of around 1000 individuals with nonoverlapping generations for five generations prior to the experiment.

The *MTP1* strain served as our stock population for wild‐type focal individuals and their potential mating partners, whereas beetles from the mutant stock ‘reindeer honey dipper’ (hereafter, *RdHD*) served as competitors during the male and female fitness assays. The *RdHD* strain is characterized by a dominant mutation affecting antenna morphology, which facilitates paternity assessment in the polygamous treatment (i.e. in the setup where *RdHD* individuals acted as competitors of wild‐type individuals). *RdHD* mutants generally show an inferior reproductive performance especially in terms of male competitiveness (Godwin et al., 2018; Sbilordo & Martin, 2014). However, all focal individuals in this experiment have been obtained from the wild‐type *MTP1* strain and the decreased reproductive performance of *RdHD* competitors is balanced across treatment groups. In our laboratory, the *RdHD* strain is kept like the *MTP1 strain* in populations of 1000 individuals with nonoverlapping generations at 30°C.

In our laboratory, located at the Terrain d'expériences of Centre d'Écologie Fonctionnelle et Évolutive in Montpellier, both strains were kept on wheat flour with 5% dry baker's yeast at 30°C in at a relative humidity of 60%. These were also the conditions of the experiment unless otherwise stated.

### 2.2 Heat stress treatment

The experiment was carried out between September and December 2020. For logistic reasons, it was run in two consecutive experimental blocks that were 1 week apart. On day 1 of each block, we transferred approximately 600 wild‐type *MTP1* and 400 *RdHD* randomly selected individuals from our stock cultures to 100 ml plastic vials filled with 50 g of flour medium in groups of 50 individuals. In those vials, individuals were supposed to lay eggs and thereby to give rise to the generation of experimental individuals (see below). Given that the sex ratio in our laboratory cultures does not deviate from 0.5 (pers. obs. TJ), we expect an even sex ratio in those groups. After 3 days of egg laying, we removed all adults and allocated half of the vials from wild‐type parents to breed focal individuals whereas the other half was selected to breed potential mating partners. Vials with developing focal individuals (i.e. all male and female individuals for which we later measured reproductive success in fitness assays; see below) were further randomly assigned to one of three thermal stress treatments: control (constantly exposed to their adapted temperature of 30°C), heatwave (exposure to a 5‐day heatwave of 40°C during adulthood prior to mating trials) and permanent heat stress (constantly exposed to 40°C during development and early adulthood; i.e. from egg laying until mating trials). On day 4, one third of the vials containing developing focal individuals were assigned to the permanent heat exposure (constant exposure to 40°C). All other vials (containing remaining wild‐type focal individuals, wild‐type mating partners and *RdHD* competitors) remained in control conditions at 30°C. Between days 21 and 25, we sexed all developed pupae and transferred them to new vials, where they were kept (within their assigned treatment condition) in same‐sex groups of 25 individuals so that they remained unmated until the mating trials. On day 39, all individuals had reached adulthood and focal beetles that have been assigned to the heatwave treatment were now exposed to 40°C for 5 days.

The thermal optimum of *T. castaneum* is considered to be about 35°C so that a 5‐day heat exposure of 40°C mimics biologically relevant heatwaves for this species (Sales et al., 2018). By contrast, permanent exposure to 40°C is more likely close to the upper critical thermal limit of *T. castaneum* and corresponds to a 5°C increase in average global surface temperature—a worst‐case scenario that is currently projected for 2100 under further increasing emissions of greenhouse gases (IPCC, 2021). In this context, the heatwave treatment is expected to compromise gamete production rate and gamete performance, particularly in males. By contrast, the permanent treatment is predicted to not only influence gamete production and performance but also to impair the entire development from egg until adulthood thereby reducing overall individual ‘condition’ of permanently stressed focal individuals.

Thermal stress treatments were achieved by placing vials with experimental individuals in temperature‐controlled egg incubators (Ovation 56 Advance; Brinsea Ltd, UK) at a relative humidity of 60%. In total, we used six incubators so that each temperature treatment was replicated in two independent environments.

### 2.3 Mating trials and fitness assays

From day 44 to 47, after completion of the thermal stress treatments, we conducted mating trials at the control temperature (30°C), with each trial lasting 7 days. Specifically, we applied a full‐factorial design and randomly allocated focal males and females from the three thermal stress treatments to two mating system treatments of either monogamous pairs or polygamous groups. Monogamous pairs consisted of two individuals: one focal individual and one wild‐type mating partner of the other sex. Polygamous groups comprised 10 individuals: one focal individual, five wild‐type potential mating partners of the other sex and four *RdHD* competitors of the same sex as the focal. There was hence an equal sex ratio in both treatments. Importantly, there was no scope for competition for mating partners and/or their gametes in monogamous pairs, whereas there was high potential for pre‐ and postcopulatory sexual selection to operate in polygamous groups. Monogamous and polygamous treatments were kept in 20 ml plastic scintillation vials filled with 5 g of flour medium so that females could lay eggs during the 7‐day trial.

We acknowledge that in our experimental design, the mating system treatment was confounded with density (density was five times higher in the polygamous groups). However, during the 7‐days lasting mating trials food was provided ad libitum to ensure that density effects on adults imposed by food limitation were negligible. Yet, other density effects on adults caused by higher encounter rates and potentially more harassment in polygamous groups during the mating trials cannot be ruled out (Michalczyk et al., 2011). In fact, individuals in monogamous pairs produced more offspring than individuals in polygamous groups (Table S1), which might have been the consequence of higher larval density and/or increased male harm under polygamy. Higher larval density in vials of polygamous groups might have led to food deprivation, which could have caused the lower number of offspring observed in the polygamy treatment. However, larvae of *T. castaneum* can develop on a very small amount of flour and we did not observe any obvious sign of food shortage in vials of polygamous groups (e.g. white flour turning into brown dust). Altogether and based on our data we cannot identify the ultimate cause for this difference in per capita offspring production between monogamous and polygamous groups. However, it is important to note that here we were not interested in comparing offspring production between monogamous or polygamous groups but on the effect of the mating system on heat stress response (i.e. the difference in reproductive success between control and heat‐stressed individuals). We hence do not expect that differences in per capita offspring production confound our results regarding the effect of the mating system on heat stress responses.

Moreover, it is important to stress that in our experimental setup only a focal individual was exposed to the heat stressed treatment, which was then challenged against unstressed competitors in the polygamy treatment. Admittedly, such a scenario is unlikely to occur in nature except for populations with substantial spatial heterogeneity in thermal conditions, for example, due to microhabitat diversity. However, our experimental setup was designed to test how sexual selection modulates net selection against maladapted phenotypes. On the assumption that there is standing genetic variation in heat resistance in a population, the applied experimental setup allows to estimate the impact of sexual selection on the strength of selection against genetic variants that are deleterious under heat stress, which is essential to predict the impact of sexual selection on the adaptation to elevated temperatures.

After the first run of mating trials, we transferred all pairs and groups to fresh vials for a second run of mating trials that also lasted 7 days, after which all individuals were discarded. We kept all vials and let all offspring (i.e. eggs laid in the medium) develop until adulthood. Afterwards, all vials were frozen with the adult offspring on day 94 of the experiment. In the following days, reproductive success of focal individuals was assayed by counting the number of adult offspring. In the monogamous treatment, all offspring had wild‐type phenotype whereas in polygamous treatment offspring could be either wild‐type or *RdHD* phenotype if one of the parents was an *RdHD* competitor (i.e. the mother was *RdHD* competitor in female assays or when sired by *RdHD* competitors in male assays). Fitness of focal individuals from monogamous treatments was quantified as the total number of wild‐type offspring. By contrast, fitness of focal individuals from polygamous treatments was quantified as the proportion of wild‐type offspring over the total number of offspring (i.e. the sum of wild‐type and *RdHD* individuals). Offspring counts from vials obtained in the first mating trials provide fitness estimates immediately after the heat stress treatment (hereafter ‘fitness assay 1’). By contrast, counts from vials of the second run of mating trials provide fitness estimates after focal individuals had the opportunity to recover from the heat stress treatment for 7 days (hereafter ‘fitness assay 2’). With this setup, we could assess reproductive success of focal individuals over two mating trials (with or without time to recover) to explore sex‐specific recovery from thermal stress under contrasting mating systems.

In total, the experiment comprised 175 focal males and 177 focal females (Table S2). Initially, we aimed for a sample size of 30 for all treatments and both sexes except for individuals of the control and heatwave treatment under monogamy for which we intended to have 40 and 50 replicates respectively. This unbalanced design was implemented because focal individuals of the monogamy treatment were planned to be used in a follow‐up experiment. Because the monogamy treatment under control temperature was identical for both sexes, we pooled the data from the replicates which we originally predefined as male and female assay (*N* = 78).

In total, we lost 68 (16.2%) replicates due to mistakes during the transfer of groups to new vials (*N* = 39; 9.3%) or because an individual of a given group died during the mating trials (*N* = 29; 6.9%). On average, we had to exclude 2.6% of monogamous pairs and 12.8% of polygamous groups because at least one individual was found dead after the mating trials. This corresponds to a five times higher likelihood to find one dead individual by chance in groups of 10 individuals (polygamy) compared to groups of two individuals (monogamy) suggesting negligible differences in per capita mortality between both mating system treatments. Moreover, mortality was slightly lower in controls (5.8%) compared to the heatwave treatment (7.1%) and the permanent heat stress treatment (8.6%) but these differences were not statistically significant (𝜒^2^ = 0.765, *df* = 2, *p* = 0.682).

### 2.4 Statistical analyses

All statistical analyses were carried out in R version 4.0.3 (R Core Team, 2021) and included two steps. First, we applied generalized linear mixed models (GLMMs) implemented in the *lme4* R‐package version 1.1.23 (Bates et al., 2015) to test for an effect of thermal stress on reproductive success separately for each sex and mating system. For the monogamous treatment, we defined the number of wild‐type offspring as the response variable assuming a Poisson error distribution with a log link function. For the polygamous treatment, we defined the number of wild‐type offspring and the number of *RdHD* offspring as response variables assuming a binomial error distribution with a logit link function. We fitted heat stress treatment as the only fixed effect together with block, incubator and observation level as random terms (effects of random terms are shown in Table S3). Observation‐level identity was added to account for overdispersion. In addition to tests for an overall treatment effect, we performed *post‐hoc* tests to evaluate pairwise differences between the three heat stress levels. In addition to the analysis of relative reproductive success under polygamy, we also report GLMMs on the absolute number of wild‐type offspring counted for the polygamy treatment in the Supporting Information (Figure S1; Table S4). Moreover, in an additional series of GLMMs we combined the data obtained from males and females and included the heat stress treatment, sex and their interaction as fixed terms to test whether sexes were differently affected by the heat stress (Table S5).

Second, we computed a selection coefficient to compare the effect of the heat stress treatment between mating systems, sexes and fitness assays. Specifically, we defined the selection coefficient (*s*) as

$$s=\frac{\left({\bar{W}}_{\text{Control}}-{\bar{W}}_{\text{Stress}}\right)}{{\bar{W}}_{\text{Control}}}=1-\left(\frac{{\bar{W}}_{\text{Stress}}}{{\bar{W}}_{\text{Control}}}\right)$$

where $\bar{W}$_Control_ is the mean number of offspring produced under control conditions (i.e. 30°C) and $\bar{W}$_Stress_ is the mean number of offspring produced under the stressed conditions (i.e. either heatwave of 40°C for 5 days or permanent exposure to 40°C). Thus, *s* is the proportional fitness loss due to thermal stress and represents a standardized effect size for the selective advantage of control individuals over heat‐stressed individual (Janicke & Chapuis, 2016; Zikovitz & Agrawal, 2013). Note that positive values of *s* indicate selection against heat‐stressed individuals whereas negative values imply that heat‐stressed individuals are favoured by selection compared to control individuals. We applied bootstrapping with 10,000 iterations using the *boot* R‐package version 1.3.28 (Canty & Ripley, 2021) to compute 95% confidence intervals for estimates of *s*. Finally, based on the obtained bootstrap samples, we computed the pairwise difference of the selection coefficient Δ*s* and its 95% confidence intervals for the comparison of sexes (Δ*s*_Sex_ = *s*_Males_ ‐ *s*_Female_), mating systems (Δ*s*_Mating system_ = *s*_Polygamy_ ‐ *s*_Monogamy_) and fitness assays (Δ*s*_Fitness assay_ = *s*_Fitness assay 1_ ‐ *s*_Fitness assay 2_). We considered estimates of Δ*s* to be significant when confidence intervals did not overlap with zero, whereas estimates centred on zero were considered to provide support for the absence of an effect.

## 3 RESULTS

**FIGURE 1:**
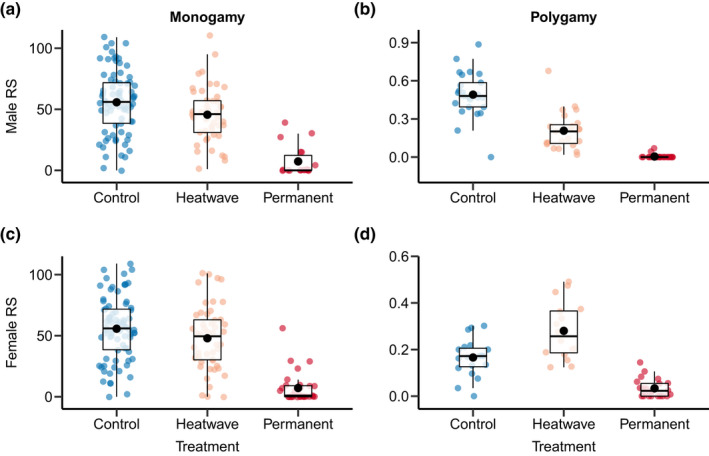
Comparison of reproductive success between heat stress treatments when measured immediately after heat exposure (‘fitness assay 1’). Boxplots are shown for males (a, b) and females (c, d) under monogamy (a, c) and polygamy (b, d). Heat stress treatment includes control (constant 30°C), heatwave (5 days of 40°C), and permanent heat stress exposure (constant 40°C). Boxplots show the 25th percentile, the median, and the 75th percentile and whiskers denote the 5th and the 95th percentiles. Filled black circles represent the means.

**FIGURE 2:**
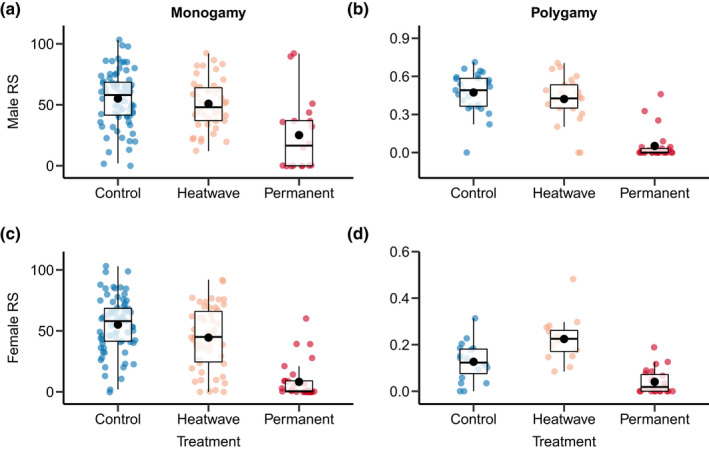
Comparison of reproductive success between heat stress treatments when measured 7 days after heat exposure (‘fitness assay 2’). Boxplots are shown for males (a, b) and females (c, d) under monogamy (a, c) and polygamy (b, d). heat stress treatment includes control (constant 30°C), heatwave (5 days of 40°C), and permanent heat stress exposure (constant 40°C). Boxplots show the 25th percentile, the median, and the 75th percentile and whiskers denote the 5th and the 95th percentiles. Filled black circles represent the means. Means together with 95% confidence intervals.

**FIGURE 3:**
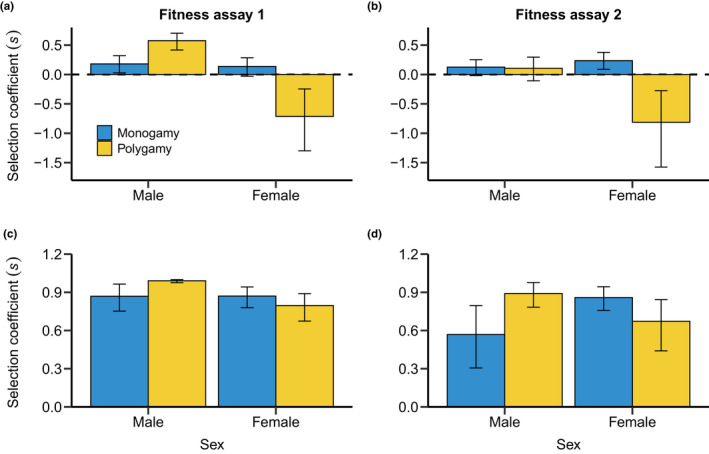
Effects of mating system on selection coefficients (s) estimating the selective advantage of control individuals over heat stressed individuals. Selection coefficients are shown for males and females under monogamy (blue) and polygamy (yellow). Comparisons shown for heatwave (a, b) and permanent heat exposure (c, d) when measured directly after the treatment (a, c; ‘fitness assay 1’) and 7 days after the treatment (b, d; ‘fitness assay 2’). Error bars denote bootstrapped means together with 95% confidence intervals.

Heat stress had a substantial impact on reproductive success (Figures 1 and 2; Tables 1 and 2), but the strength of observed effects highly depended on the sex, mating system and fitness assay (Figure 3; Tables 3 and 4). For males, heatwaves impaired male reproductive success when facing competition in the polygamy setting (i.e. a reduction of 57.7% in reproductive success in fitness assay 1), but only tended to have a detrimental effect under monogamy (Figure 1a and b; Table 1), which manifested via a significant effect of the mating system (Figure 3; Table 3). The expected negative effect of heatwave on male reproductive success could only be detected immediately after heatwave exposure, but not after 7 days of recovery (i.e. a reduction of 10.9%; fitness assay 2: Figure 2; Table 2). In contrast to our findings on the effects of heatwave, permanent heat exposure had a consistent, negative impact across both mating systems (Table 1). Still, similar to the heatwave pattern, the negative effect of permanent heat exposure was stronger under polygamy than under monogamy (i.e. a reduction of male reproductive success in the polygamy treatment of 99.1% in fitness assay 1 and 89.1% in fitness assay 2; Figure 3; Table 4). Importantly, permanent heat stress exposure had a negative effect on males not only immediately after the treatment but also beyond 7 days of recovery (Figure 2; Table 2). However, the negative effects were found to be weaker in fitness assay 2 suggesting partial recovery of male reproductive performance (Table 4).

**TABLE 1:** Effect of heat stress on male and female reproductive success immediately after the heat stress treatments (‘fitness assay 1’) under monogamy (i.e. one male and one female) and polygamy (i.e. five males and five females). Table shows results obtained from generalized linear mixed models testing for an overall treatment effect and all pairwise comparisons between control (constant 30°C), heatwave (5 days of 40°C) and permanent heat exposure (constant 40°C). Statistically significant effects are marked in boldface

Mating system	Sex	Treatment effect			Post‐hoc comparisons	Estimate	*SE*	*z*‐value	Adj. *p*‐value*
		*N*	*χ* ^2^	*p*‐value	Contrast				
Monogamy	Male	141	119.280	**<0.001**	Control ‐ Heatwave	−0.207	0.118	−1.755	0.079
					Control ‐ Permanent	2.818	0.259	10.886	**<0.001**
					Heatwave ‐ Permanent	2.611	0.270	9.669	**<0.001**
	Female	158	153.245	**<0.001**	Control ‐ Heatwave	−0.217	0.160	−1.356	0.175
					Control ‐ Permanent	−2.745	0.225	−12.190	**<0.001**
					Heatwave ‐ Permanent	−2.528	0.237	−10.674	**<0.001**
Polygamy	Male	73	135.537	**<0.001**	Control ‐ Heatwave	−1.456	0.318	−4.583	**<0.001**
					Control ‐ Permanent	−6.223	0.538	−11.564	**<0.001**
					Heatwave ‐ Permanent	−4.766	0.542	−8.802	**<0.001**
	Female	58	95.012	**<0.001**	Control ‐ Heatwave	0.838	0.325	2.582	**0.010**
					Control ‐ Permanent	−1.980	0.321	−6.174	**<0.001**
					Heatwave ‐ Permanent	−2.818	0.296	−9.511	**<0.001**

* Raw *p*‐values adjusted to account for false discovery rate using the method by Benjamini and Hochberg (1995).

**TABLE 2:** Effect of heat stress on male and female reproductive success 7 days after the heat stress treatments (‘fitness assay 2’) under monogamy (i.e. one male and one female) and polygamy (i.e. five males and five females). Table shows results obtained from generalized linear mixed models testing for an overall treatment effect and all pairwise comparisons between control (constant 30°C), heatwave (5 days of 40°C) and permanent heat exposure (constant 40°C). Statistically significant effects are marked in boldface

Mating system	Sex	Treatment effect			Post‐hoc comparisons	Estimate	*SE*	*z*‐value	Adj. *p*‐value*
		*N*	*χ* ^2^	*p*‐value	Contrast				
Monogamy	Male	141	51.837	**<0.001**	Control ‐ Heatwave	−0.067	0.149	−0.449	0.654
					Control ‐ Permanent	−1.511	0.218	−6.946	**<0.001**
					Heatwave ‐ Permanent	−1.444	0.220	−6.556	**<0.001**
	Female	158	148.097	**<0.001**	Control ‐ Heatwave	−0.370	0.169	−2.185	**0.029**
					Control ‐ Permanent	−2.855	0.236	−12.098	**<0.001**
					Heatwave ‐ Permanent	−2.484	0.248	−10.009	**<0.001**
Polygamy	Male	73	96.953	**<0.001**	Control ‐ Heatwave	−0.270	0.399	−0.676	0.499
					Control ‐ Permanent	−4.388	0.476	−9.225	**<0.001**
					Heatwave ‐ Permanent	−4.118	0.478	−8.609	**<0.001**
	Female	58	52.996	**<0.001**	Control ‐ Heatwave	0.952	0.426	2.234	**0.025**
					Control ‐ Permanent	−1.563	0.428	−3.652	**<0.001**
					Heatwave ‐ Permanent	−2.515	0.347	−7.242	**<0.001**

* Raw *p*‐values adjusted to account for false discovery rate using the method by Benjamini and Hochberg (1995)

**TABLE 3:** Effect of heatwave treatment on male and female reproductive success. Table shows bootstrapped selection coefficients together with 95% intervals (in parentheses) estimating the selective advantage of control individuals (constant 30°C) over heat stressed individuals (5 days of heatwave of 40°C). Positive values indicate selection against heat‐stressed individuals. Bootstrapped differences Δ*s* between mating systems, sexes and fitness assays are marked in boldface whenever confidence intervals do not include zero

Fitness assay	Mating system	Male	Female	Δ*s*_Sex_
Fitness assay 1	Monogamy	0.179 (0.024, 0.319)	0.138 (−0.024, 0.288)	0.041 (−0.176, 0.258)
	Polygamy	0.575 (0.424, 0.700)	−0.714 (−1.317, −0.248)	**1.289 (0.802, 1.906)**
	Δ*s*_Mating system_	**0.396 (0.190, 0.593)**	**−0.852 (−1.466, −0.353)**	
Fitness assay 2	Monogamy	0.124 (−0.018, 0.252)	0.233 (0.080, 0.375)	−0.109 (−0.311, 0.086)
	Polygamy	0.102 (−0.106, 0.290)	−0.818 (−1.585, −0.289)	**0.920 (0.339, 1.722)**
	Δ*s*_Mating system_	−0.022 (−0.268, 0.217)	**−1.051 (−1.823, −0.489)**	
Δ*s*_Fitness assay_	Monogamy	0.055 (−0.146, 0.253)	−0.095 (−0.313, 0.118)	
	Polygamy	**0.473 (0.237, 0.718)**	0.104 (−0.707, 0.993)	

**TABLE 4:** Effect of permanent heat exposure on male and female reproductive success. Table shows bootstrapped selection coefficients *s* together with 95% intervals (in parentheses) estimating the selective advantage of control individuals (constant 30°C) over heat‐stressed individuals (permanent heat exposure to 40°C). Bootstrapped differences Δ*s* between mating systems, sexes and fitness assays are marked in boldface whenever confidence intervals do not include zero

Fitness assay	Mating system	Male	Female	Δ*s*_Sex_
Fitness assay 1	Monogamy	0.870 (0.755, 0.964)	0.870 (0.782, 0.941)	−0.001 (−0.138, 0.129)
	Polygamy	0.991 (0.976, 1.000)	0.795 (0.676, 0.890)	**0.196 (0.099, 0.315)**
	Δ*s*_Mating system_	**0.121 (0.025, 0.236)**	−0.075 (−0.214, 0.055)	
Fitness assay 2	Monogamy	0.568 (0.304, 0.795)	0.859 (0.759, 0.941)	**−0.291 (−0.567, −0.047)**
	Polygamy	0.890 (0.776, 0.976)	0.673 (0.447, 0.842)	**0.216 (0.012, 0.457)**
	Δ*s*_Mating system_	**0.322 (0.074, 0.604)**	−0.186 (−0.428, 0.013)	
Δ*s*_Fitness assay_	Monogamy	**0.302 (0.050, 0.585)**	0.012 (−0.108, 0.136)	
	Polygamy	**0.101 (0.015, 0.215)**	0.122 (−0.089, 0.372)	

In females, heatwave had a weak negative effect on reproductive success under monogamy, which was found to be statistically significant only in fitness assay 2 (i.e. a reduction of 14.0% in fitness assay 1 and of 23.6% in fitness assay 2; Figure 1C; Table 1). In the polygamy treatment, however, we observed a higher reproductive output of females exposed to the heatwave (Figure 1D; Table 1). Thus, the mating system had a strong effect on heatwave response in females, changing its direction (Figure 3; Table 3). By contrast, permanent heat exposure had a negative effect under both mating systems (Figure 2C and D). This negative effect was generally stronger in the monogamy treatment, but was not found to be significantly different between mating systems in both fitness assays (Table 4). Overall, we found that the observed effects of heatwave and permanent heat exposure on females remained largely constant over the two fitness assays (Tables 3 and 4).

Finally, heat stress also had sex‐specific effects. Overall, we found support that the effect of heat stress differed between males and females under polygamy but not under monogamy (Table S5). The comparison of the selection coefficients confirmed this pattern. Specifically, we observed that under polygamy, both heat stress treatments had a stronger negative effect on males than on females (Tables 3 and 4).

## 4 DISCUSSION

Our study provides compelling evidence for the role of sexual selection as a moderator of sex‐specific heat stress responses in the form of four main findings. First, heat stress affected reproductive performance of males and females. Second, mating systems altered heat stress responses in both sexes. Remarkably, the observed effect of sexual selection on heatwave responses was found to have opposing directions for males and females. Third, males showed full recovery from heatwave and partial recovery from permanent heat stress exposure. By contrast, beneficial and detrimental effects on females had long‐term effects with no signs of deterioration or recovery respectively. And fourth, the sex difference in heat response depended on the mating system. While heat stress had a similar effect on males and females in the monogamy setting, we observed a stronger sensitivity to heat stress in males under polygamy.

### 4.1 Effect of heat stress on male and female reproductive success

For males, we detected a nonsignificant, negative effect of heatwave in the monogamy treatment but a reduction of 57.6% in reproductive success under polygamy, that is, under conditions allowing for pre‐ and postcopulatory sexual selection to operate. Therefore, the presence of sexual selection is associated with detrimental effects of heat stress on males. Permanent heat exposure impaired male reproductive success drastically in both mating systems, but similar to our findings on heatwaves, the effect was magnified in the polygamy treatment in which we observed an almost complete reproductive failure (i.e. a reduction of 99.1%). Overall, these findings are consistent with results of a previous study demonstrating a negative effect of a 5‐day heatwave on male sperm competitiveness inferred from assays in which focal males were paired with an already mated female (Sales et al., 2018). Interestingly, Sales et al. (2018) also investigated the underlying mechanisms causing the observed heat response, and showed that heatwaves act by lowering mating rates and reducing both sperm numbers and sperm viability.

Similar to our results, Sales et al. (2018) and Sales et al. (2021) observed a reduction of male reproductive success of 21.3% after heatwaves of 40°C under monogamy. We detected a comparable but statistically nonsignificant tendency for a reduction of male reproductive success of (first fitness assay: 18.3% reduction; second fitness assay: 21.8%). For females, we observed a lowered reproductive success of individuals experiencing the heatwave treatment (first fitness assay: 14.2% reduction; second fitness assay: 23.6%) and those experiencing permanent thermal stress (first fitness assay: 87.1% reduction; second fitness assay: 85.9%) under monogamy. However, the negative effect of heatwave exposure was found to be statistically significant only in the second fitness assay. These findings also largely correspond to results of previous studies that did not detect significant differences between females exposed to 30°C and 40°C arguing that *T. castaneum* has its thermal optimum at 35°C (Sales et al., 2018; Sales et al., 2021). Nevertheless, other studies on *T. castaneum* reported a lowered female fecundity under monogamy at 35°C or 37°C compared to 30°C or 33°C respectively (Fischer et al., 2021; Koch & Guillaume, 2020).

Presumably the most surprising finding of our study is the observed positive net effect of heatwave on female reproductive success under polygamy. Even though permanent heat exposure had a negative effect on female reproductive success under polygamy, its magnitude was lower compared to the negative effect detected under monogamy. Thus, our findings suggest that females may benefit from having access to multiple partners under elevated temperatures. Experimental studies testing for fitness consequences of polyandry in *T. castaneum* provided mixed results, but overall suggest that females obtain indirect rather than direct benefits (Bernasconi & Keller, 2001; Matsumura et al., 2021; Pai et al., 2005; Pai et al., 2007; Pai & Yan, 2002; Pai & Yan, 2020). As such, the question of how potential benefits of polyandry depend on the thermal conditions remains largely unexplored. Interestingly, Grazer and Martin (2012) exposed female *T. castaneum* to ambient temperatures of 30°C versus 34°C and observed an increase in reproductive success at elevated temperatures under polygamy but not under monogamy—similar to our findings. They argued that seminal fluids may mitigate detrimental effects of temperature and desiccation stress (Edvardsson, 2007; Poiani, 2006), because they contain important substances such as nutrients and water, that are a limiting factor for female reproductive success, particularly under elevated temperatures (Grazer & Martin, 2012). Red flour beetles only acquire water indirectly via air humidity and food, but not directly by drinking. Hence, polygamous females may benefit from having access to multiple mating partners under elevated temperatures by receiving more water‐ and nutrient‐rich spermatophores as documented in other insects such as bruchid beetles (Edvardsson, 2007) and bush‐crickets (Gwynne & Simmons, 1990). However, to fully explain our findings on female reproductive success, heat‐stressed females do not only need to obtain direct (or indirect) benefits from having access to multiple partners but also need to have a higher mating rate compared to females exposed to control temperatures. Alternatively, the increased reproductive success observed in heatwave exposed females, might have been related to sexual conflict. In *Drosophila*, the transfer of seminal fluid proteins has been found to cause a short‐term boost of egg laying after mating at the expense of future reproductive success (Hollis et al., 2019). Similar phenomena of sexual conflict may also occur in Tribolium beetles (Fedina & Lewis, 2008), which is expected to elicit counteradaptations in females to resist male manipulation. Importantly, heat‐stressed females might be more prone to suffer from male manipulation due to a lowered capacity to counteract male manipulation, which would translate into a higher short‐term female reproductive output of heatwave exposed individuals compared to control individuals. Further work is clearly required to identify the underlying processes explaining the observed interaction between thermal stress and mating system. Moreover, our results illustrate the need to study heat response in females not only under monogamy but also in settings allowing for sexual selection to operate. There is growing evidence that sexual selection is a potent evolutionary force in females (Fromonteil et al., 2021; Hare & Simmons, 2019) but whether it has an effect on female stress responses is very poorly understood.

Finally, our study also highlights an important aspect regarding the temporal persistence of the observed effects. Specifically, we found evidence that males have the capacity to recover from heat stress (fully or partly depending on the duration of the heat stress treatment), whereas effects on females remained constant along the tested time window of 2 weeks after heat exposure. These findings largely coincide with findings by Sales et al. (2021) who detected full recovery of male reproductive performance 25 days after heat exposure.

### 4.2 Effect of sexual selection on sex‐specific heat response

Our study demonstrates that polygamy triggers a negative heat response in males but relaxes detrimental effects of heat stress in females. This striking finding suggests that sexual selection can reverse the sex differences in heat sensitivity. Thermal stress has often been argued to have stronger effects on males compared to females but experimental studies in arthropods testing for sex‐specific heat responses provided mixed results ranging from a male‐bias (Sales et al., 2018; Walsh et al., 2021) to no sex differences (Piyaphongkul et al., 2012) and female‐biased thermal sensitivity (Janowitz & Fischer, 2011). Our results suggest that mating system might be an important determinant of intra‐ and interspecific variation of sex differences in heat sensitivity. In a recent study, Baur et al. (2022) tested for a short‐term evolutionary response in heat sensitivity under varying mating systems in the seed beetle *Callosobruchus maculatus*. Their findings suggest that populations evolving under polygamy show a higher sensitivity to heat stress in females compared to males when reproductive success was assayed under monogamy. Thus, our findings together with the results from Baur et al. (2022) indicate that mating system can be a potent driver for sex differences in heat response at the phenotypically plastic and micro‐evolutionary scale.

Sex differences in heat response are important to project the adaptive responses of populations to increased temperatures, which is a pressing question in the face of current global warming and increased frequency of extreme climatic events. Theory predicts that sexual selection can accelerate adaptation to changing environments when two conditions are fulfilled (Holman & Kokko, 2013; Whitlock & Agrawal, 2009). First, sexual selection and natural selection need to be aligned meaning that sexual selection favours genetic variants that are also beneficial under natural selection (Rowe & Rundle, 2021). Second, net selection must be stronger on males compared to females so that populations can purge deleterious alleles at a low demographic cost (Whitlock & Agrawal, 2009; Winkler et al., 2021). In *T. castaneum*, there is compelling evidence for an alignment of natural and sexual selection (Godwin et al., 2020; Lumley et al., 2015), however, it remains to be tested whether alleles that are beneficial under thermal stress in males are also advantageous under thermal stress in females. Our study suggests that sexual selection (i.e. polygamy) intensifies net selection on heat resistance in males but not in females. Hence, adaptation to increased temperatures may indeed be mediated by sexual selection on males as documented for other arthropods (Parrett & Knell, 2018; Plesnar‐Bielak et al., 2012).

## 5 CONCLUSIONS

Our findings demonstrate that the intensity of sexual selection plays a pivotal role in predicting heat responses in both sexes. We found that polygamy magnifies detrimental effects of heat stress in males but potentially relaxes negative effects on females. Thus, sexual selection may promote a sex difference in thermal sensitivity and thereby the sex‐specific strength of selection on alleles associated with heat resistance. A better understanding of how populations adapt to rising temperatures and increased frequency of extreme climatic events will critically depend on more detailed knowledge on the thermal optima under varying levels of sexual selection. Importantly, experimental studies testing for an effect of sexual selection on heat responses should not only focus on males but also include females. Finally, quantitative genetics and genomic approaches have the potential to examine whether heat stress favours the same genetic variants in male and females, which is essential to evaluate the role of sexual selection for modulating adaptation to climate change.

## AUTHOR CONTRIBUTIONS

Tim Janicke conceived the study. Tim Janicke, Maria Moiron, Lennart Winkler and Oliver Yves Martin designed the study. Tim Janicke and Maria Moiron collected the data. Tim Janicke ran the statistical analyses. Tim Janicke and Maria Moiron wrote the paper with the help of Oliver Yves Martin and Lennart Winkler.

## CONFLICT OF INTEREST

All authors declare no conflict of interest.

## Supporting information


Figure S1
Table S1
Table S2
Table S3
Table S4
Table S5

## Data Availability

Data available from the Dryad Digital Repository https://doi.org/10.5061/dryad.98sf7m0n7 (Moiron et al., 2022).

## References

[fec14204-bib-0001] Angilletta, M. J. (2009). Thermal adaptation: A theoretical and empirical synthesis. Oxford University Press, Inc.

[fec14204-bib-0002] Bates, D. , Maechler, M. , Bolker, B. , & Walker, S. (2015). Fitting linear mixed‐effects models using lme4. Journal of Statistical Software, 67, 1–48.

[fec14204-bib-0003] Baur, J. , Jagusch, D. , Michalak, P. , Koppik, M. , & Berger, D. (2022). The mating system affects the temperature sensitivity of male and female fertility. Functional Ecology, 36, 92–106.

[fec14204-bib-0004] Benjamini, Y. , & Hochberg, Y. (1995). Controlling the false discovery rate ‐ a practical and powerful approach to multiple testing. Journal of the Royal Statistical Society Series B: Statistical Methodology, 57, 289–300.

[fec14204-bib-0005] Bernasconi, G. , & Keller, L. (2001). Female polyandry affects their sons' reproductive success in the red flour beetle *Tribolium castaneum* . Journal of Evolutionary Biology, 14, 186–193. 29280574 10.1046/j.1420-9101.2001.00247.x

[fec14204-bib-0006] Bonduriansky, R. , Maklakov, A. , Zajitschek, F. , & Brooks, R. (2008). Sexual selection, sexual conflict and the evolution of ageing and life span. Functional Ecology, 22, 443–453.

[fec14204-bib-0007] Canty, A. , & Ripley, B. D. (2021). boot: Bootstrap R (S‐Plus) functions . R package version 1.3‐28.

[fec14204-bib-0008] Coumou, D. , & Rahmstorf, S. (2012). A decade of weather extremes. Nature Climate Change, 2, 491–496.

[fec14204-bib-0009] David, P. , Bjorksten, T. , Fowler, K. , & Pomiankowski, A. (2000). Condition‐dependent signalling of genetic variation in stalk‐eyed flies. Nature, 406, 186–188. 10910358 10.1038/35018079

[fec14204-bib-0010] De Lisle, S. P. (2019). Understanding the evolution of ecological sex differences: Integrating character displacement and the Darwin‐Bateman paradigm. Evolution Letters, 3, 434–447.

[fec14204-bib-0011] Edvardsson, M. (2007). Female *Callosobruchus maculatus* mate when they are thirsty: Resource‐rich ejaculates as mating effort in a beetle. Animal Behaviour, 74, 183–188.

[fec14204-bib-0012] Fedina, T. Y. , & Lewis, S. M. (2008). An integrative view of sexual selection in Tribolium flour beetles. Biological Reviews, 83, 151–171. 18429767 10.1111/j.1469-185X.2008.00037.x

[fec14204-bib-0013] Fischer, E. M. , & Knutti, R. (2015). Anthropogenic contribution to global occurrence of heavy‐precipitation and high‐temperature extremes. Nature Climate Change, 5, 560–564.

[fec14204-bib-0014] Fischer, K. , Kreyling, J. , Beaulieu, M. , Beil, I. , Bog, M. , Bonte, D. , Holm, S. , Knoblauch, S. , Koch, D. , Muffler, L. , Mouginot, P. , Paulinich, M. , Scheepens, J. F. , Schiemann, R. , Schmeddes, J. , Schnittler, M. , Uhl, G. , van der Maaten‐Theunissen, M. , Weier, J. M. , … Gienapp, P. (2021). Species‐specific effects of thermal stress on the expression of genetic variation across a diverse group of plant and animal taxa under experimental conditions. Heredity, 126, 23–37. 32632284 10.1038/s41437-020-0338-4 PMC7852598

[fec14204-bib-0015] Fromonteil, S. , Winkler, L. , Marie‐Orleach, L. , & Janicke, T. (2021). Sexual selection in females across the animal tree of life. *bioRxiv*, 10.1101/2021.1105.1125.445581 PMC9831318 36626380

[fec14204-bib-0016] Garcia‐Roa, R. , Garcia‐Gonzalez, F. , Noble, D. W. A. , & Carazo, P. (2020). Temperature as a modulator of sexual selection. Biological Reviews, 95, 1607–1629. 32691483 10.1111/brv.12632

[fec14204-bib-0017] Godwin, J. L. , Lumley, A. J. , Michalczyk, Ł. , Martin, O. Y. , & Gage, M. J. (2020). Mating patterns influence vulnerability to the extinction vortex. Global Change Biology, 26, 4226–4239. 32558066 10.1111/gcb.15186

[fec14204-bib-0018] Godwin, J. L. , Spurgin, L. G. , Michalczyk, L. , Martin, O. Y. , Lumley, A. J. , Chapman, T. , & Gage, M. J. G. (2018). Lineages evolved under stronger sexual selection show superior ability to invade conspecific competitor populations. Evolution Letters, 2, 511–523. 30283698 10.1002/evl3.80 PMC6145403

[fec14204-bib-0019] Grazer, V. M. , & Martin, O. Y. (2012). Elevated temperature changes female costs and benefits of reproduction. Evolutionary Ecology, 26, 625–637.

[fec14204-bib-0020] Gwynne, D. T. , & Simmons, L. W. (1990). Experimental reversal of courtship roles in an insect. Nature, 346, 172–174.

[fec14204-bib-0021] Hämäläinen, A. , Immonen, E. , Tarka, M. , & Schuett, W. (2018). Evolution of sex‐specific pace‐of‐life syndromes: Causes and consequences. Behavioral Ecology and Sociobiology, 72, 1–15. 10.1007/s00265-018-2462-1 PMC5856903 29576676

[fec14204-bib-0022] Hare, R. M. , & Simmons, L. W. (2019). Sexual selection and its evolutionary consequences in female animals. Biological Reviews, 94, 929–956. 30484943 10.1111/brv.12484

[fec14204-bib-0023] Hollis, B. , Koppik, M. , Wensing, K. U. , Ruhmann, H. , Genzoni, E. , Erkosar, B. , Kawecki, T. J. , Fricke, C. , & Keller, L. (2019). Sexual conflict drives male manipulation of female postmating responses in *Drosophila melanogaster* . Proceedings of the National Academy of Sciences of the United States of America, 116, 8437–8444. 30962372 10.1073/pnas.1821386116 PMC6486729

[fec14204-bib-0024] Holman, L. , & Kokko, H. (2013). The consequences of polyandry for population viability, extinction risk and conservation. Philosophical Transactions of the Royal Society B: Biological Sciences, 368, 12. 10.1098/rstb.2012.0053 PMC3576587 23339244

[fec14204-bib-0025] Huey, R. B. , Kearney, M. R. , Krockenberger, A. , Holtum, J. A. M. , Jess, M. , & Williams, S. E. (2012). Predicting organismal vulnerability to climate warming: Roles of behaviour, physiology and adaptation. Philosophical Transactions of the Royal Society B: Biological Sciences, 367, 1665–1679. 10.1098/rstb.2012.0005 PMC3350654 22566674

[fec14204-bib-0026] IPCC . (2021). The physical science basis. Contribution of Working Group I to the Sixth Assessment Report of the Intergovernmental Panel on Climate Change .

[fec14204-bib-0027] Janicke, T. , & Chapuis, E. (2016). Condition‐dependence of male and female reproductive success: Insights from a hermaphrodite. Ecology and Evolution, 6, 830–841. 26865970 10.1002/ece3.1916 PMC4739575

[fec14204-bib-0028] Janicke, T. , Häderer, I. K. , Lajeunesse, M. J. , & Anthes, N. (2016). Darwinian sex roles confirmed across the animal kingdom. Science Advances, 2, e1500983. 26933680 10.1126/sciadv.1500983 PMC4758741

[fec14204-bib-0029] Janowitz, S. A. , & Fischer, K. (2011). Opposing effects of heat stress on male versus female reproductive success in *Bicyclus anynana* butterflies. Journal of Thermal Biology, 36, 283–287.

[fec14204-bib-0030] Jones, A. G. , & Ratterman, N. L. (2009). Mate choice and sexual selection: What have we learned since Darwin? Proceedings of the National Academy of Sciences of the United States of America, 106, 10001–10008. 19528643 10.1073/pnas.0901129106 PMC2702796

[fec14204-bib-0031] Koch, E. L. , & Guillaume, F. (2020). Additive and mostly adaptive plastic responses of gene expression to multiple stress in *Tribolium castaneum* . PLoS Genetics, 16, e1008768. 32379753 10.1371/journal.pgen.1008768 PMC7238888

[fec14204-bib-0032] Lovegrove, B. (2014). Cool sperm: Why some placental mammals have a scrotum. Journal of Evolutionary Biology, 27, 801–814. 24735476 10.1111/jeb.12373

[fec14204-bib-0033] Lumley, A. J. , Michalczyk, L. , Kitson, J. J. N. , Spurgin, L. G. , Morrison, C. A. , Godwin, J. L. , Dickinson, M. E. , Martin, O. Y. , Emerson, B. C. , Chapman, T. , & Gage, M. J. G. (2015). Sexual selection protects against extinction. Nature, 522, 470–473. 25985178 10.1038/nature14419

[fec14204-bib-0034] Matsumura, K. , Miyatake, T. , & Yasui, Y. (2021). An empirical test of the bet‐hedging polyandry hypothesis: Female red flour beetles avoid extinction via multiple mating. Ecology and Evolution, 11, 5295–5304. 34026007 10.1002/ece3.7418 PMC8131809

[fec14204-bib-0035] Michalczyk, L. , Millard, A. L. , Martin, O. Y. , Lumley, A. J. , Emerson, B. C. , & Gage, M. J. G. (2011). Experimental evolution exposes female and male responses to sexual selection and conflict in *Tribolium castaneum* . Evolution, 65, 713–724. 21091981 10.1111/j.1558-5646.2010.01174.x

[fec14204-bib-0036] Moiron, M. , Winkler, L. , Martin, O. Y. & Janicke, T. (2022). Sexual selection moderates heat stress response in males and females. *Dryad Digital Repository*, 10.5061/dryad.5098sf5067m5060n5067 PMC10092254 37064077

[fec14204-bib-0037] Moore, C. R. (1926). The biology of the mammalian testis and scrotum. Quarterly Review of Biology, 1, 4–50.

[fec14204-bib-0038] Pai, A. , Bennett, L. , & Yan, G. Y. (2005). Female multiple mating for fertility assurance in red flour beetles (*Tribolium castaneum*). Canadian Journal of Zoology, 83, 913–919.

[fec14204-bib-0039] Pai, A. , Feil, S. , & Yan, G. (2007). Variation in polyandry and its fitness consequences among populations of the red flour beetle, *Tribolium castaneum* . Evolutionary Ecology, 21, 687–702.

[fec14204-bib-0040] Pai, A. D. , & Yan, G. Y. (2020). Long‐term study of female multiple mating indicates direct benefits in *Tribolium castaneum* . Entomologia Experimentalis et Applicata, 168, 398–406.

[fec14204-bib-0041] Pai, A. T. , & Yan, G. Y. (2002). Polyandry produces sexy sons at the cost of daughters in red flour beetles. Proceedings of the Royal Society B: Biological Sciences, 269, 361–368. 10.1098/rspb.2001.1893 PMC1690898 11886623

[fec14204-bib-0042] Parmesan, C. (2006). Ecological and evolutionary responses to recent climate change. Annual Review of Ecology Evolution and Systematics, 37, 637–669.

[fec14204-bib-0043] Parratt, S. R. , Walsh, B. S. , Metelmann, S. , White, N. , Manser, A. , Bretman, A. J. , Hoffmann, A. A. , Snook, R. R. , & Price, T. A. (2021). Temperatures that sterilize males better match global species distributions than lethal temperatures. Nature Climate Change, 11, 481–484.

[fec14204-bib-0044] Parrett, J. M. , & Knell, R. J. (2018). The effect of sexual selection on adaptation and extinction under increasing temperatures. Proceedings of the Royal Society B: Biological Sciences, 285, 7. 10.1098/rspb.2018.0303 PMC5936732 29669902

[fec14204-bib-0045] Pilakouta, N. , & Ålund, M. (2021). Sexual selection and environmental change: What do we know and what comes next? Current Zoology, 67, 293–298. 34616921 10.1093/cz/zoab021 PMC8488989

[fec14204-bib-0046] Piyaphongkul, J. , Pritchard, J. , & Bale, J. (2012). Can tropical insects stand the heat? A case study with the brown planthopper *Nilaparvata lugens* (Stål). PLoS One, 7, e29409. 22253720 10.1371/journal.pone.0029409 PMC3257224

[fec14204-bib-0047] Plesnar‐Bielak, A. , Skrzynecka, A. M. , Prokop, Z. M. , & Radwan, J. (2012). Mating system affects population performance and extinction risk under environmental challenge. Proceedings of the Royal Society B: Biological Sciences, 279, 4661–4667. 10.1098/rspb.2012.1867 PMC3479737 22977151

[fec14204-bib-0048] Poiani, A. (2006). Complexity of seminal fluid: A review. Behavioral Ecology and Sociobiology, 60, 289–310.

[fec14204-bib-0049] R Core Team . (2021). A language and environment for statistical computing. R Foundation for Statistical Computing. http://www.R‐project.org/

[fec14204-bib-0050] Rowe, L. , & Houle, D. (1996). The lek paradox and the capture of genetic variance by condition dependent traits. Proceedings of the Royal Society of London Series B: Biological Sciences, 263, 1415–1421.

[fec14204-bib-0051] Rowe, L. , & Rundle, H. D. (2021). The alignment of natural and sexual selection. Annual Review of Ecology, Evolution, and Systematics, 52, 499–517.

[fec14204-bib-0052] Sales, K. , Vasudeva, R. , Dickinson, M. E. , Godwin, J. L. , Lumley, A. J. , Michalczyk, L. , Hebberecht, L. , Thomas, P. , Franco, A. , & Gage, M. J. G. (2018). Experimental heatwaves compromise sperm function and cause transgenerational damage in a model insect. Nature Communications, 9, 11. 10.1038/s41467-018-07273-z PMC6233181 30425248

[fec14204-bib-0053] Sales, K. , Vasudeva, R. , & Gage, M. J. (2021). Fertility and mortality impacts of thermal stress from experimental heatwaves on different life stages and their recovery in a model insect. Royal Society Open Science, 8, 201717. 33959335 10.1098/rsos.201717 PMC8074959

[fec14204-bib-0054] Sbilordo, S. H. , & Martin, O. Y. (2014). Pre‐ and postcopulatory sexual selection act in concert to determine male reproductive success in *Tribolium castaneum* . Biological Journal of the Linnean Society, 112, 67–75.

[fec14204-bib-0055] Stoehr, A. M. , & Kokko, H. (2006). Sexual dimorphism in immunocompetence: What does life‐history theory predict? Behavioral Ecology, 17, 751–756.

[fec14204-bib-0056] Tarka, M. , Guenther, A. , Niemelä, P. T. , Nakagawa, S. , & Noble, D. W. (2018). Sex differences in life history, behavior, and physiology along a slow‐fast continuum: A meta‐analysis. Behavioral Ecology and Sociobiology, 72, 1–13. 10.1007/s00265-018-2534-2 PMC6060830 30100667

[fec14204-bib-0057] Tattersall, G. J. , Sinclair, B. J. , Withers, P. C. , Fields, P. A. , Seebacher, F. , Cooper, C. E. , & Maloney, S. K. (2012). Coping with thermal challenges: Physiological adaptations to environmental temperatures. Comprehensive Physiology, 2, 2151–2202. 23723035 10.1002/cphy.c110055

[fec14204-bib-0058] Walsh, B. S. , Mannion, N. L. , Price, T. A. , & Parratt, S. R. (2021). Sex‐specific sterility caused by extreme temperatures is likely to create cryptic changes to the operational sex ratio in *Drosophila virilis* . Current Zoology, 67, 341–343. 34616928 10.1093/cz/zoaa067 PMC8489007

[fec14204-bib-0059] Walsh, B. S. , Parratt, S. R. , Hoffmann, A. A. , Atkinson, D. , Snook, R. R. , Bretman, A. , & Price, T. A. (2019). The impact of climate change on fertility. Trends in Ecology & Evolution, 34, 249–259. 30635138 10.1016/j.tree.2018.12.002

[fec14204-bib-0060] Whitlock, M. C. , & Agrawal, A. F. (2009). Purging the genome with sexual selection: Reducing mutation load through selection on males. Evolution, 63, 569–582. 19154364 10.1111/j.1558-5646.2008.00558.x

[fec14204-bib-0061] Winkler, L. , Moiron, M. , Morrow, E. H. , & Janicke, T. (2021). Stronger net selection on males across animals. eLife, 10, e68316. 34787569 10.7554/eLife.68316 PMC8598160

[fec14204-bib-0062] Zikovitz, A. E. , & Agrawal, A. F. (2013). The condition dependency of fitness in males and females: The fitness consequences of juvenile diet assessed in environments differing in key adult resources. Evolution, 67, 2849–2860. 24094338 10.1111/evo.12170

[fec14204-bib-0063] Zorgniotti, A. W. (1991). Temperature and environmental effects on the testis. Springer.

