# Supplementary material for: Sexual selection moderates heat stress response in males and females
Source: Funct Ecol. 2022 Oct 27;36(12):3096–106. doi: 10.1111/1365-2435.14204 (PMC10092254; doi:10.1111/1365-2435.14204)
Supplement: Supplementary file 1 — Figure S1 Table S1 Table S2 Table S3 Table S4 Table S5 [file FEC-36-3096-s001.docx]

Supporting Information for

**Sexual selection moderates heat stress response in males and females**

Maria Moiron, Lennart Winkler, Oliver Yves Martin, Tim Janicke*

* Corresponding author (tim.janicke@cefe.cnrs.fr)

This file includes:

Figure S1.

Table S1 – S5.

**Figure S1. Comparison of male and female reproductive success (RS) between heat stress treatments measured under polygamy.** Values of absolute offspring numbers are shown for fitness assay 1 (A, C) and fitness assay 2 (B, D). Boxplots are shown for males (A, B) and females (C, D). Heat stress treatment includes control (constant 30 °C), heatwave (five days of 40 °C), and permanent heat stress exposure (constant 40 °C). Boxplots show the 25th percentile, the median, and the 75th percentile and whiskers denote the 5th and the 95th percentiles. Filled black circles represent the means.

**Table S1. Comparison of female reproductive success between monogamous and polygamous groups.** Table shows sample sizes (*N*), means and standard errors (SE) of the number of offspring produced by focal females under monogamy and polygamy. Test statistics obtained from Generalized Linear Mixed-Effects Models including mating system as fixed effect and block and incubator as random terms. Statistically significant effects are marked in boldface.

| Fitness assay | Treatment | Monogamy | | | Polygamy | | | 𝜒^2^-value | *P*-value |
| --- | --- | --- | --- | --- | --- | --- | --- | --- | --- |
|  |  | *N* | Mean | SE | *N* | Mean | SE |  |  |
| 1^st^ | Control | 78 | 55.72 | 2.93 | 19 | 18.21 | 2.03 | 74.789 | **< 0.001** |
|  | Heatwave | 50 | 47.90 | 3.70 | 16 | 29.31 | 2.15 | 2.975 | 0.085 |
|  | Permanent | 30 | 7.17 | 2.27 | 23 | 3.26 | 0.74 | 0.030 | 0.862 |
| 2^nd^ | Control | 78 | 58.23 | 3.07 | 19 | 11.47 | 2.24 | 92.226 | **< 0.001** |
|  | Heatwave | 50 | 44.48 | 3.64 | 16 | 22.94 | 2.24 | 9.294 | **0.002** |
|  | Permanent | 30 | 8.20 | 2.71 | 23 | 3.22 | 0.96 | 0.112 | 0.738 |

**Table S2. Overview of sample sizes.** Number of focal male and female individuals (*N*) in each mating system and heat stress treatments.

| Mating System | Temperature | Sex | *N* |
| --- | --- | --- | --- |
| Monogamy | Control | Male and female | 78 |
|  | Heatwave | Male | 45 |
|  |  | Female | 50 |
|  | Permanent | Male | 18 |
|  |  | Female | 30 |
| Polygamy | Control | Male | 25 |
|  |  | Female | 19 |
|  | Heatwave | Male | 23 |
|  |  | Female | 16 |
|  | Permanent | Male | 25 |
|  |  | Female | 23 |

**Table S3. Effects of random terms (block and incubator) on male and female reproductive success shown for both fitness assays (1 and 2) and mating systems (monogamy and polygamy).** Table shows results obtained from Generalized Linear Mixed-Effects Models testing for an overall treatment effect (see Main Text for fixed effects).

| Fitness assay | Mating system | Sex | Term | *df* | *χ^2^* | *P*-value |
| --- | --- | --- | --- | --- | --- | --- |
| Fitness assay 1 | Monogamy | Male | Block | 1 | 0.004 | 0.951 |
|  |  |  | Incubator | 1 | 0.004 | 0.951 |
|  |  | Female | Block | 1 | < 0.001 | > 0.999 |
|  |  |  | Incubator | 1 | < 0.001 | > 0.999 |
|  | Polygamy | Male | Block | 1 | < 0.001 | > 0.999 |
|  |  |  | Incubator | 1 | < 0.001 | > 0.999 |
|  |  | Female | Block | 1 | 1.099 | 0.295 |
|  |  |  | Incubator | 1 | 0.365 | 0.546 |
| Fitness assay 2 | Monogamy | Male | Block | 1 | 5.650 | 0.017 |
|  |  |  | Incubator | 1 | 0.117 | 0.733 |
|  |  | Female | Block | 1 | 8.643 | 0.003 |
|  |  |  | Incubator | 1 | < 0.001 | 0.999 |
|  | Polygamy | Male | Block | 1 | 0.124 | 0.724 |
|  |  |  | Incubator | 1 | < 0.001 | > 0.999 |
|  |  | Female | Block | 1 | < 0.001 | > 0.999 |
|  |  |  | Incubator | 1 | 1.039 | 0.308 |

**Table S4. Effect of heat stress on the absolute number of offspring produced by males and females shown for both fitness assays** **under polygamy (i.e., five males and five females).** Table shows results obtained from Generalized Linear Mixed-Effects Models testing for an overall treatment effect and all pair-wise comparisons between control (constant 30 °C), heatwave (five days of 40 °C) and permanent heat exposure (constant 40 °C). Statistically significant effects are marked in boldface.

| Fitness assay | Sex | Treatment effect | | | Post-hoc comparisons |  |  |  |  |
| --- | --- | --- | --- | --- | --- | --- | --- | --- | --- |
|  |  | *N* | 𝜒^2^ | *P*-value | Contrast | Estimate | *SE* | *z*-value | adj. *P*-value^*^ |
| Fitness assay 1 | Male | 73 | 136.494 | **< 0.001** | Control - Heatwave | -0.968 | 0.232 | -4.167 | **< 0.001** |
|  |  |  |  |  | Control - Permanent | -4.113 | 0.441 | -9.335 | **< 0.001** |
|  |  |  |  |  | Heatwave - Permanent | 5.082 | 0.438 | 11.613 | **< 0.001** |
|  | Female | 58 | 110.455 | **< 0.001** | Control - Heatwave | 0.557 | 0.218 | 2.552 | **0.011** |
|  |  |  |  |  | Control - Permanent | -2.354 | 0.233 | -10.121 | **< 0.001** |
|  |  |  |  |  | Heatwave - Permanent | 1.797 | 0.227 | 7.924 | **< 0.001** |
| Fitness assay 2 | Male | 73 | 96.249 | **< 0.001** | Control - Heatwave | -0.456 | 0.308 | -1.478 | 0.140 |
|  |  |  |  |  | Control - Permanent | -3.163 | 0.385 | -8.222 | **< 0.001** |
|  |  |  |  |  | Heatwave - Permanent | 3.619 | 0.381 | 9.501 | **< 0.001** |
|  | Female | 58 | 49.147 | **< 0.001** | Control - Heatwave | 1.022 | 0.364 | 2.805 | **0.005** |
|  |  |  |  |  | Control - Permanent | -2.485 | 0.356 | -6.974 | **< 0.001** |
|  |  |  |  |  | Heatwave - Permanent | 1.463 | 0.363 | 4.025 | **< 0.001** |

^*^raw *P*-values adjusted to account for false discovery rate using the method by Benjamini and Hochberg (1995)

**Table S5. Effect of heat stress and sex on reproductive success under monogamy and polygamy shown for both fitness assays.** Table shows results obtained from Generalized Linear Mixed-Effects Models testing for an overall heat stress effect, sex effect and their interaction. Statistically significant effects are marked in boldface.

| Fitness assay | Mating System | *N* | Treatment effect | | Sex effect | | Treatment by sex interaction | |
| --- | --- | --- | --- | --- | --- | --- | --- | --- |
|  |  |  | 𝜒^2^-value | *P*-value | 𝜒^2^-value | *P*-value | 𝜒^2^-value | *P*-value |
| Fitness assay 1 | Monogamy | 221 | 222.927 | **< 0.001** | 1.567 | 0.211 | 1.754 | 0.416 |
|  | Polygamy | 131 | 162.178 | **< 0.001** | 0.999 | 0.318 | 57.808 | **< 0.001** |
| Fitness assay 2 | Monogamy | 221 | 160.355 | **< 0.001** | 3.703 | 0.054 | 14.080 | **0.001** |
|  | Polygamy | 131 | 116.399 | **< 0.001** | 16.187 | **< 0.001** | 15.697 | **< 0.001** |

# References

Benjamini, Y. & Hochberg, Y. (1995) Controlling the false discovery rate - a practical and powerful approach to multiple testing. *Journal of the Royal Statistical Society Series B-Statistical Methodology,* **57,** 289-300.
